# Methadone rotation versus other opioid rotation for refractory cancer induced bone pain: protocol of an exploratory randomised controlled open-label study

**DOI:** 10.1186/s12904-023-01160-1

**Published:** 2023-04-15

**Authors:** Natasha Michael, Merlina Sulistio, Robert Wojnar, Alexandra Gorelik

**Affiliations:** 1 Supportive, Psychosocial and Palliative Care Research Department, Cabrini Health, Malvern Victoria, Australia; 2 School of Medicine, Sydney Campus, University of Notre Dame Australia Darlinghurst, Darlinghurst, NSW Australia; 3 Faculty of Medicine, Nursing and Health Sciences, Monash University, Melbourne Victoria, Australia; 4 Department of Epidemiology and Preventative Medicine, Alfred Hospital, Melbourne Victoria, Australia

**Keywords:** Cancer pain, Opioids, Methadone, Bone pain, Opioid rotation

## Abstract

**Background:**

A third of patients with advanced cancer and bone metastasis suffer from cancer induced bone pain (CIBP), impeding quality of life, psychological distress, depression and anxiety. This study will evaluate the impact of an opioid rotation, comparing methadone rotation with other opioid rotation in patients with refractory CIBP.

**Methods:**

This open-label randomised controlled trial will recruit cancer patients with CIBP and inadequate pain control despite established baseline opioid and/or intolerable opioid side effects from cancer and palliative care services. Participants will be at least 18 years old, with a predicted prognosis of greater than 8 weeks, meet the core diagnostic criteria for CIBP, have a worst pain score of ≥ 4 of 10 from CIBP and/ or have opioid toxicity (graded ≥ 2 on Common Terminology Criteria for Adverse Events). Participants will have sufficiently proficient English to complete questionnaires and provide informed consent.

Participants will be randomised 1:1 to be rotated to methadone to another opioid. The primary objective is to examine the impact of opioid rotation in improving CIBP by comparing analgesic efficacy, safety and tolerability in the two arms. Secondary objectives will assess changes in the intensity, duration and frequency of breakthrough pain, requirement of breakthrough analgesia, overall opioid escalation index, and time taken to observe improvement in pain reduction, pain interference and quality of life.

**Discussion:**

Laboratory studies suggest the involvement of neuropathic involvement in the mechanism of CIBP, though there remains no clear evidence of the routine use of neuropathic agents. Methadone as an analgesic agent may have a role to play in this cohort of patients, thus warranting further exploratory studies.

**Trial Registration:**

Australian New Zealand Clinical Trials Registry No: ACTRN12621000141842. Registered 11 February 2021.

**Supplementary Information:**

The online version contains supplementary material available at 10.1186/s12904-023-01160-1.

## Background

Cancer induced bone pain (CIBP) is a common cancer pain syndromes with complex pathophysiology, comprising a mix of inflammatory nociceptive and neuropathic pain pathways [1]. Approximately two-thirds of patients with advanced cancer suffer from varying degrees of bone pain, with the most common bone metastases arising from multiple myeloma, cancer of the breast, prostate, lungs, thyroid, kidneys, and ovaries [2].

The characteristics of CIBP, with mixed mechanism pain and the combination of background and breakthrough (spontaneous and incident) pain leads to the challenges with its management, necessitating a multimodal approach [1]. Current treatments for CIBP include the use of pharmaceuticals such bisphosphonates and anti-RANK-L antibodies, opioids and non-opioid co-analgesics such as non-steroidal anti-inflammatories, corticosteroids, antidepressants and anticonvulsants in conjunction with radiotherapy, radio-isotopes and interventional procedures [1, 3].

Despite increasing knowledge in the pathophysiology and mechanism of CIBP, there has been limited translation into clinical practice to guide the choice of available analgesic treatments [4]. Laboratory studies suggest the involvement of neuropathic involvement in the mechanism of pain, though there remains no clear evidence of the routine use of neuropathic agents [3, 4]. Cancer pain typically presents with a significant neuropathic element [5], therefore the challenge remains as how to distinguish neuropathic pain arising from CIBP from that of overall cancer pain. A recent multi-centre, double-blind, randomized trial of pregabalin versus placebo in 233 patients with CIBP showed no statistically significant difference in average pain or pain interference between both arms [6]. Thus opioids remain the mainstay treatment for CIBP, despite animal modelling revealing a degree of opioid resistance [1] and no evidence to guide the choice of opioids [4].

Our department’s clinical experience in the use of racemic methadone as an alternative opioid in patients with refractory CIBP unresponsive to morphine or other opioids or those who exhibit dose limiting toxicity has not been substantiated by evidence. Our use of methadone is influenced by its known antagonistic effects at the N-methyl-D-aspartate (NMDA) receptor channel and inhibition of the reuptake of serotonin and noradrenaline [7]. Our retrospective study of 94 patients rotated to methadone for refractory CIBP demonstrated that 70% and 53% of patients achieved a ≥ 30% and ≥ 50% reduction in pain respectively, with mean pain intensity reduced from 5.6 (SD = 2.1) to 2.6 (SD = 2.5), *p* < 0.001 [8]. On completion of the methadone rotation (MR), over 70% of patients required an actual lower dose of methadone compared to their predicted/ calculated daily methadone dose (mean 25.7 mg (SD = 10.9) vs 17.0 mg (SD = 8.5)). The mean number of breakthrough opioid analgesia used a day reduced from 3.4 (SD = 2.3) to 1.8 (SD = 1.7), *p* < 0.001. These results provide preliminary evidence that methadone for refractory CIBP might provide benefits, but this requires confirmation by further exploratory studies as at this point there have been no other randomised controlled trial evidence for methadone in CIBP.

In this study we define refractory cancer pain as cancer pain that does not respond to standard opioid and/or co-analgesic therapy [9]. This study aims to examine the impact of opioid rotation to methadone in improving CIBP compared to other opioid rotation (OOR). We hypothesise that compared to OOR, MR in patients suffering from refractory CIBP will result in improvement in pain control with a better tolerated side effect profile. We will compare analgesic efficacy, safety and tolerability of a MR compared to OOR in patients with CIBP.

The secondary aim is to assess changes in the intensity, duration and frequency of breakthrough pain, requirement of breakthrough analgesia, overall opioid escalation index, time taken to observe improvement in pain reduction, pain interference and patient’s quality of life (QOL). We will additionally assess for correlations between trough levels of methadone at the end of the study period and analgesic response in the MR arm. The protocol is outlined according to the Standard Protocol Items: Recommendations for Interventional Trials guidelines [10].

## Methods / design

### Study design and setting

This is an exploratory, single site, open-label, randomised controlled trial with two parallel arms enrolling 50 cancer patients already established on a strong opioid with refractory CIBP. Refractory CIBP is defined as worst pain score rated at ≥ 4 of 10 on a numeric rating scale (NRS) [11] and/or current opioid analgesic resulting in opioid toxicity (defined by grade ≥ 2 on Common Terminology Criteria for Adverse Events Version 5.0, (CTCAE) (Additional file 1) [12]. Patients will be rotated from their usual opioid to racemic methadone or from usual opioid to another strong opioid (morphine, oxycodone or hydromorphone) based on best practice guidelines [13]. Participants will be followed up for 14 days following rotation.

### Participants and recruitment

The study will be conducted at an 850 bed metropolitan hospital in Melbourne, Australia. Patients with a solid tumour or haematological cancer diagnosis with a predicted prognosis of greater than 8 weeks will be recruited from the oncology and palliative services. Eligible patients 1) will be at least 18 years old; 2) have confirmed bone metastasis, with CIBP being the dominant pain. Bone metastasis will be confirmed through radiological investigations and patients must meet the core diagnostic criteria for CIBP as defined by The Analgesic, Anesthetic, and Addiction Clinical Trial Translations, Innovations, Opportunities, and Networks-American Pain Society (ACTTION-APS) (Table 1) [14]; 3) patients must already be established on a strong baseline opioid (Step 3 of WHO ladder) pre-enrolment [15] but continue to report a worst pain score from the site with CIBP of ≥ 4 of 10 on a 0–10 numeric rating scale (NRS) and/ or present with opioid toxicity (CTCAE grade ≥ 2) [12] resulting from their existing opioid. Participants with pain arising from other sites e.g. liver metastasis causing visceral pain will be eligible for the study but we will only be recording sites where CIBP are reported and all pain assessments will pertain to the CIBP.

**Table 1:** Core diagnostic criteria for cancer induced bone pain**:** ACTTION-APS^a^ [14]

1. History of primary or metastatic bone cancer diagnosed using imaging and physical examination
2. Presence of continuous, background pain (usually described as annoying, dull, gnawing, aching, and/or nagging) in 1 or more locations *generally consistent* with known distribution of bone lesions
3. Presence of evoked or spontaneous pain (often described as electric or shock-like) in 1 or more locations generally consistent with known distribution of bone lesions, associated with weight bearing or movement or can occur spontaneously
4. Clinical examination over the site of pain reveals: • Hyperalgesia to blunt, non-noxious pressure or pin-prick stimuli • Hypoesthesia to non-noxious thermal stimuli • Hypoesthesia to light touch stimuli

^a^Analgesic, Anesthetic, and Addiction Clinical Trial Translations, Innovations, Opportunities, and Networks-American Pain Society

Nerve conduction velocity and diagnostic electromyography will not be used as part of the diagnostic criteria for the neuropathic element of CIBP as the diagnosis will be made purely on clinical assessment as stated in Table 1 below.

Participants will be excluded if they received radiotherapy within 1 week of enrolment, have a QTc > 500 ms on an electrocardiogram [16], are not sufficiently proficient in English to be able to complete questionnaires and provide informed consent or deemed unsuitable to participate in the study for clinical reasons as determined by the treating physician.

### Study procedures

**Fig. 1:**
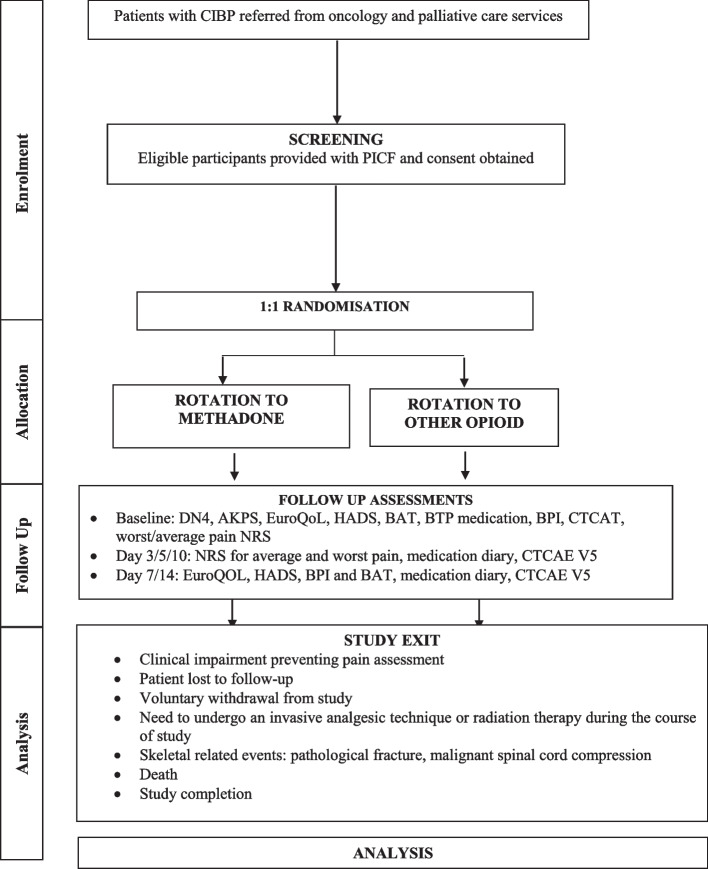
Study Procedure

The study procedure is shown in Fig. 1. Referring clinicians will refer possible eligible patients to study investigators. Patients will be screened against the study inclusion and exclusion criteria and eligible patients will be provided with a participant information and consent form. Agreeable patients will sign a consent form with the study investigator and in-patient admission for ambulatory patients will be organised.

### Randomisation

The study will be conducted in an in-patient setting. Participants will be randomised by an independent randomization administrator to either MR or OOR in a 1:1 ratio using a computer-generated random number sequence with varied block sizes of 2, 4 and 6. Concealed allocation was used using opaque numbered envelopes that were sealed (closed and glued), stored in a locked drawer and accessed only at the point of randomisation. The participant will be enrolled and allocated by the clinician researchers.

As this is an early exploratory study with a limited budget, participants will not be blinded to the interventions. Investigators conducting follow up assessments are also not blinded to the intervention to facilitate dose titration to mitigate the risk of toxicity.

## Study arms

### Methadone rotation

MR will be implemented using the rapid conversion Stop-and-Go method [16, 17]. The pre-MR long acting opioids will be converted to oral morphine equivalent daily dose (OMEDD) using established opioid conversion ratios [17]. The calculated daily dose of oral methadone (DDOM) will be obtained using published morphine-methadone conversion guidelines [16]. The prescribed DDOM on MR initiation (Day 1) will be determined following clinician assessment; taking into consideration medication interactions, hepatic impairment, the potential for tolerance to previous opioids and physiological changes affecting volume distribution, particularly if the calculated DDOM exceeds 30 mg [16–18]. Pre-MR long acting opioid will be ceased, and racemic methadone administered in three or four divided doses. The dosing and frequency of methadone administration will be adjusted according to clinical effect and observed toxicity, with the aim of twice or thrice daily dosing for improved adherence on discharge. Methadone dose adjustment will be limited to ≤ 5 mg/ day following recommended practice [16].

### Other opioid rotation

Opioid rotation with opioids other than methadone will be implemented based on established opioid conversion ratios [17]. Clinicians who are study investigators will decide which opioid (morphine, oxycodone or hydromorphone) to switch to depending on participant opioid history, allergy profile and hepatic and renal function. We will use clinician discretion when considering dose reduction on rotation as per established guidelines. Opioid dose escalation will occur as per clinical indication during the study period following rotation and will be guided by clinician judgement.

In both intervention arms, participants will continue to use immediate release and/or rapid onset opioids (not methadone) for breakthrough analgesia. There will be no limitations set on the number of breakthrough analgesics used. Use of adjuvant co-analgesic medications such as NSAID, trazodone, acetaminophen, or Tramadol [15] are permitted but titration of these medications will be restricted during the study period to ensure the observed changes in pain intensity is attributable only to the study intervention. Breakthrough medications administration daily will be obtained from the in-patient electronic medication chart or patient recording on the provided medication diary. Adjustment to laxatives and other drugs used for usual symptom benefit are permitted.

Participants will remain as an in-patient until stable opioid dose is achieved (a stable dose is defined when worst pain intensity is less than 4/10, with acceptable adverse effects (CTCAE grade < 2). Following discharge, participants will be followed up for a total of 14 days from initiation of study intervention. All follow up assessments will be conducted face to face or over the telephone.

### Data collection and measurement tools

Data will be sourced from the clinical records, electronic prescribing records and directly from participants. Baseline and ongoing data collection are summarised in Table 2.

**Table 2:** Measures used

	**Eligibility**	**Baseline Day 0**	**Day 3**	**Day 5**	**Day 7**	**Day 10**	**Day 14**
Hematology and Biochemistry screen (FBC/LFT/U&E)	** × **						x
ACTTION Criteria	** × **						
ECG	** × **						
***Reviews***							
Clinical review—doctor	** × **						
Clinical review—nurse		×	×	×	×	×	×
***Measures/Assessmens***							
DN4		** × **					
AKPS		** × **					
EuroQOL		** × **			** × **		** × **
HADS		** × **			** × **		** × **
BAT		** × **			** × **		** × **
Daily BTP medication assessment		** × **	×	×	** × **	×	** × **
BPI		×			×		×
CTCAE		×	×	×	×	×	×
Worst Pain NRS		×	×	×	×	×	×
Average Pain NRS		×	×	×	×	×	×
Semi-structured interview				×			

*ACTTION* Analgesic, anesthetic, and addiction clinical trial translations, innovations, opportunities, and networks American pain society, *ECG* Electrocardiogram, *DN4* Douleur Neuropathique 4, *AKPS* Australian Karnofsky Performance Status, *HADS* Hospital Anxiety and Depression Scale, *BAT* Breakthrough Pain Assessment Tool, *BTP* Breakthrough pain, *BPI* Brief Pain Inventory, *CTCAE* Common Terminology Criteria for Adverse Events, *NRS* Numeric Rating Scale

### Baseline data

We will collect the following data to ensure baseline comparability:

- basic demographics (age, gender, primary cancer diagnosis and sites of known bone metastases).
- current analgesic use e.g. baseline opioid use converted to OMEDD, breakthrough and co-analgesic use
- pain types (e.g. continuous/background pain, evoked/spontaneous incident pain, neuropathic) and performance status.
- history of oncology-specific therapy, radiotherapy, denosumab and bisphosphonate use.

### Ongoing data collection will occur using the following validated instruments

- a) Douleur Neuropathique 4 (DN4) is a screening tool for neuropathic pain consisting of interview questions (DN4-interview) and physical tests. A score of ≥ 4/10 suggests neuropathic pain [19].
- b) EuroQol Thermometer measures health status, ranging from worst to best possible health state [20].
- c) Hospital Anxiety and Depression Scale (HADS) is a self-rating scale to assess psychological distress in non-psychiatric patient [21].
- d) Australia-Modified Karnofsky Performance Scale (AKPS) measures the patient’s overall performance status or ability to perform their activities of daily living [22].
- e) Breakthrough pain Assessment Tool (BAT) assess the frequency, duration, severity and effect of breakthrough analgesia on breakthrough pain [23].
- f) Brief Pain Inventory (BPI) assesses pain interferences and patient satisfaction with pain relief [24].
- g) Numerical Rating Scale (NRS)** (**0-no pain to 10-pain as bad as can be imagined) is used to rate pain on a defined scale [11].
- h) Common Terminology Criteria for Adverse Events (CTCAE) grades for opioid toxicities, evaluating for somnolence, respiratory depression, confusion, hallucinations, nausea, vomiting, constipation, pruritus/ itching, and dry mouth [12].
- i) Routine blood tests and trough methadone levels

We collected routine haematology and biochemistry screens for all patients. We additionally elected to measure the trough level of methadone, which indicates the lowest concentration of methadone in the body after the drug has been broken down and metabolized by the body. Through levels are measured immediately before the next dose is given. Studies have indicated that for optimum efficacy, methadone trough levels of about 400–500 ng/ml are required [25]. We thus collected a minimum of 2mls of whole blood in a fluoride oxalate blood collection tube, between 30 min and 1 h before the next dose was due.

### Primary outcome

The primary outcome will be changes in worst and average pain intensity on day 14. A significant response is classified as ≥ 30% pain reduction and substantial response as ≥ 50% pain reduction. The proportion of responders in each group will be calculated.

### Secondary outcomes

The following outcomes will be compared between the two intervention arms:

- a) Breakthrough pain intensity, duration and frequency
- b) Frequency of breakthrough analgesia administration at each time point (averaged over the preceding 3-days)
- c) Treatment safety and tolerability using composite CTCAE scores
- d) Time taken to observe improvement will be calculated as the difference between baseline data and first date with ≥ 30% pain reduction.
- e) Opioid escalation index (OEI%) [26], a surrogate marker of opioid responsiveness will be calculated using the following formula at day 14:
  $$\frac{\frac{Total\;daily\;dose\;at\;day\;14-Total\;Daily\;dose\;at\;day\;1}{Total\;daily\;dose\;at\;day\;1}}{14}\times100$$
- f) Changes in EuroQOL, HADS, total pain interferences score and patient satisfaction with pain relief.
- g) Correlation between analgesic response trough methadone levels at day 14.

### Study failure/withdrawal

Patients who developed severe adverse reactions likely to be secondary to MR or OOR, such as delirium will have their treatment discontinued and alternative analgesics prescribed as per usual clinical processes. Patients who develop complications unrelated to treatment such as pathological fractures or malignant spinal cord compression and those who require invasive analgesic techniques or radiation therapy during the study will be withdrawn. Data will.continued to be collected up to the point of withdrawal and included in the analysis plan. Alladverse events will be reported to the trail monitoring committee and participants will beoffered opportunities to seek support for any adverse outcome.

### Sample size and power

This is an exploratory study. We chose to recruit 50 participants (25 per arm) to this study based on the absence of any preliminary data related to timing to pain reduction and change in pain intensity for those treated with methadone and assuming that methadone is equivalent to other opioids in relation to the expected proportion of patients reaching > 30% or 50% pain reduction [27]. Additionally, the sample size estimations were based on similar studies [28], and based on variance minimisation stratagem [29]. This sample size will be feasible in terms of recruitment, will have 80% power to detect a large treatment effect (Cohen’s d = 0.8) and also provide the ability to adjust the analysis for potential baseline differences between study arms.

### Statistical analysis

The summary statistics will be reported as mean (SD) or median (IQR) for continuous data and N (%) for categorical data. The results of all regression models will be reported as point estimate with 95%CI as appropriate. Level of significance was mentioned as a standard procedure, but all results will be interpreted with respect to both statistical significance and clinical relevance/importance. Between group differences will be assessed using either Student T-test or Wilcoxon rank-sum test for continuous data or either Chi2 or Fisher’s exact test for categorical variables. Additionally, standardised difference between two groups will be calculated and values > 0.1 will be indicative of between group imbalances.

A mixed effect model will be used to assess the longitudinal differences in pain reduction, opioids escalation and quality of life measures between methadone and other opioids groups. Survival analysis will be used to compare time to event outcomes (i.e. time taken to observe pain improvement). Kaplan-Meyer survival curves will be constructed and either log-rank test or Cox proportional hazard model will be used. Spearman correlation will be used to examine any correlations between reduction in pain intensity and QOL score, HADS and other relevant continuous outcomes.

As stated in the study protocol, all data collected until patient’s withdrawal from the study or death will be included in the data analysis and for the basis for the primary analysis.

The sensitivity analysis for the primary outcome will be considered, subject to study completion rate, and will be performed using LOCF, worst/best case scenario imputations. This will be detailed in the final manuscript of the completed randomised controlled trial as appropriate. The analysis will be performed using Stata16 [30] and *p* < 0.05 will be considered statistically significant for all tests.

## Quality standards

### Randomization and blinding

In view of logistical and financial considerations, this study will be an open-label study with both participants and study investigator/ clinicians administering the intervention being aware of treatment allocation. Research statistician and research support staff assisting with data entry will be blinded to allocation.

Following randomisation, the clinical trial pharmacist will be notified to ensure dispensing of the appropriate medication. The participant ID, date of request, preparation and dispensing will be recorded in a log maintained by the site pharmacist for each randomisation.

### Staff training

Clinicians and research staff involved in the study will be trained on screening and approaching patients for recruitment, consent, data collection, coding and storage. Staff will also be trained on identifying risk, managing adverse effects and how to deal with any distress that the participants may display whilst completing the study and how to refer appropriately for support.

### Data monitoring and confidentiality

A data monitoring committee will include an independent pain clinician, pharmacist and.researcher to provide ongoing oversight into early results and ensure adherence to the research protocol. Data will be stored in a secure database with information and measurements stored independently from identifiable personal information to ensure confidentiality. Findings of the study will be presented at national and international meetings and published in peer reviewed journals.

## Limitations

This study is limited by it being a single site, non-blinded study with a small sample size. The research team’s exploratory work identified the lack of use of methadone as a primary analgesic in many potential collaborative sites (most use it as an adjunct analgesic). It is hoped that this preliminary work will increase the confidence of sites in considering enrolment in a subsequent multi-site study. Additionally, it is difficult in cancer pain studies to limit other confounding factors such as radiotherapy and the use of bisphosphonates that may influence pain outcomes and as such a pragmatic approach was used in the study design.

## Conclusion

CIBP continues to be a source of much suffering for cancer patients, warranting exploration of novel treatment options. Methadone as an analgesic agent may have a role to play in this cohort of patients, thus warranting further exploratory studies.

## Supplementary Information

**Additional file 1: Appendix 1. **Common Terminology Criteria for Adverse Events: Opioid Toxicity Assessment Version 5.0. 

## Data Availability

Not applicable. Data sharing is not applicable as this article has no datasets that have been generated or analysed yet. The datasets generated during the study will be available from the corresponding author on reasonable request.

## Abbreviations

ACTTION-APS: Analgesic, Anesthetic, and Addiction Clinical Trial Translations, Innovations, Opportunities, and Networks-American Pain Society
AKPS: Australian-modified Karnofsky Performance Scale
BAT: Breakthrough pain Assessment Tool
BTP: Breakthrough Pain
BPI: Brief Pain Inventory
CI: Confidence Interval
CIBP: Cancer Induced Bone Pain
CTCAE: Common Terminology Criteria for Adverse Events
DN4: Douleur Neuropathique 4 questions
ECG: Electrocardiography
HREC: Human Research Ethics Committee
IQR: Interquartile range
MR: Methadone Rotation
NRS: Numerical Rating Score
OEI: Opioid Escalation Index
OOR: Other Opioid Rotation
PI: Principal Investigator
QOL: Quality of Life
SD: Standard Deviation
WHO: World Health Organisation

## References

[1] Kane CM, Hoskin P, Bennett MI (2015). Cancer induced bone pain. BMJ.

[2] Coleman RE (2001). Metastatic bone disease: clinical features, pathophysiology and treatment strategies. Cancer Treat Rev.

[3] Middlemiss T, Laird BJ, Fallon MT (2011). Mechanisms of cancer-induced bone pain. Clin Oncol.

[4] Zajaczkowska R, Kocot-Kepska M, Leppert W, Wordliczek J (2019). Bone pain in cancer patients: mechanisms and current treatment. Int J Mol Sci.

[5] Davis MP (2018). Cancer-related neuropathic pain: review and selective topics. Hematol Oncol Clin North Am.

[6] Fallon M, Hoskin PJ, Colvin LA, Fleetwood-Walker SM, Adamson D, Byrne A (2016). Randomized double-blind trial of Pregabalin versus placebo in conjunction with palliative radiotherapy for cancer-induced bone pain. J Clin Oncol.

[7] McPherson  ML, Walker KA, Davis MP, Reddy A, Paice J (2019). Safe and appropriate use of methadone in hospice and palliative care: Expert Consensus White Pape. J Pain Symptom Manage.

[8] Sulistio M, Wojnar R, Key S, Kwok J, Al-Rubaie Z, Michael N (2021). The role of methadone in cancer-induced bone pain: a retrospective cohort study. Support Care Cancer.

[9] Afsharimani B, Kindl K, Good P (2015). Pharmacological options for the management of refractory cancer pain—what is the evidence?. Support Care Cancer.

[10] Chan AW, Tetzlaff JM, Altman DG, Laupacis A, Gøtzsche PC, Krleža-Jerić K (2013). SPIRIT 2013 statement: defining standard protocol items for clinical trials. Ann Intern Med.

[11] Thong ISK, Jensen MP, Miro J, Tan G (2018). The validity of pain intensity measures: what do the NRS, VAS, VRS, and FPS-R measure?. Scand J Pain.

[12] Basch E, Becker C, Rogak LJ, Schrag D, Reeve BB, Spears P (2021). Composite grading algorithm for the National Cancer Institute's Patient-Reported Outcomes version of the Common Terminology Criteria for Adverse Events (PRO-CTCAE). Clin Trials.

[13] Caraceni A, Hanks G, Kaasa S, Bennett MI, Brunelli C, Cherny N (2012). Use of opioid analgesics in the treatment of cancer pain: evidence-based recommendations from the EAPC. Lancet Oncol.

[14] Paice JA, Mulvey M, Bennett M, Dougherty PM, Farrar JT, Mantyh PW (2017). AAPT diagnostic criteria for chronic cancer pain conditions. J Pain.

[15] Fallon M, Giusti R, Aielli F, Hoskin P, Rolke R, Sharma M (2018). Management of cancer pain in adult patients: ESMO Clinical Practice Guidelines. Ann Oncol.

[16] McLean S, Twomey F (2015). Methods of rotation from another strong opioid to methadone for the management of cancer pain: a systematic review of the available evidence. J Pain Symptom Manage.

[17] Mercadante S, Caraceni A (2011). Conversion ratios for opioid switching in the treatment of cancer pain: a systematic review. Palliat Med.

[18] Lugo RA, Satterfield KL, Kern SE (2005). Pharmacokinetics of methadone. J Pain Palliat Care Pharmacother.

[19] Timmerman H, Steegers MAH, Huygen F, Goeman JJ, van Dasselaar NT, Schenkels MJ, Wilder-Smith OHG, Wolff AP, Vissers KCP (2017). Investigating the validity of the DN4 in a consecutive population of patients with chronic pain. PLoS ONE.

[20] EuroQol Group (1990). EuroQol–a new facility for the measurement of health-related quality of life. Health Policy.

[21] LoMartire R, Ang BO, Gerdle B, Vixner L (2020). Psychometric properties of Short Form-36 Health Survey, EuroQol 5-dimensions, and Hospital Anxiety and Depression Scale in patients with chronic pain. Pain.

[22] Abernethy AP, Shelby-James T, Fazekas BS, Woods D, Currow DC (2005). The Australia-modified Karnofsky Performance Status (AKPS) scale: a revised scale for contemporary palliative care clinical practice. BMC Palliat Care.

[23] Webber K, Davies AN, Zeppetella G, Cowie MR (2014). Development and validation of the breakthrough pain assessment tool (BAT) in cancer patients. J Pain Symptom Manage.

[24] Cleeland CS, Ryan KM (1994). Pain assessment: global use of the Brief Pain Inventory. Ann Acad Med Singapore.

[25] Westermeyer JJ, Yoon G, Thuras P, Batres-Y-Carr T, Dickmann P (2016). Pharmacotherapy in methadone maintenance: Clinical utility of peak-trough blood levels. Addict Disord Their Treat.

[26] Mercadante S, Dardanoni G, Salvaggio L, Armata MG, Agnello A (1997). Monitoring of opioid therapy in advanced cancer pain patients. J Pain Symp Manage.

[27] Mercadante S, Porzio G, Ferrera P, Fulfaro F, Aielli F, Verna L (2008). Sustained-release oral morphine versus transdermal fentanyl and oral methadone in cancer pain management. Eur J Pain.

[28] Cubero DI, del Giglio A (2010). Early switching from morphine to methadone is not improved by acetaminophen in the analgesia of oncologic patients: a prospective, randomized, double-blind, placebo-controlled study. Support Care Cancer.

[29] Whitehead AL, Julious SA, Cooper CL, Campbell MJ (2016). Estimating the sample size for a pilot randomised trial to minimise the overall trial sample size for the external pilot and main trial for a continuous outcome variable. Stat Methods Med Res.

[30] StataCorp.  (2019). Stata Statistical Software: Release 16.

