# Supplementary material for: Methadone rotation versus other opioid rotation for refractory cancer induced bone pain: protocol of an exploratory randomised controlled open-label study
Source: BMC Palliat Care. 2023 Apr 15;22:42. doi: 10.1186/s12904-023-01160-1 (PMC10105403; doi:10.1186/s12904-023-01160-1)
Supplement: Supplementary file 1 — Additional file 1: Appendix 1. Common Terminology Criteria for Adverse Events: Opioid Toxicity Assessment Version 5.0. [file 12904_2023_1160_MOESM1_ESM.docx]

**Appendix 1: Common Terminology Criteria for Adverse Events: Opioid Toxicity Assessment Version 5.0**

Have you experienced any side effects from your pain management?

Prompt for list of side effects below if not readily volunteered. Grade side effects according to table below and enter in AE and concurrent medication log.

**Grade 1** - Mild; asymptomatic or mild symptoms; clinical or diagnostic observations only; intervention not indicated.

**Grade 2 -** Moderate; minimal, local or non-invasive intervention indicated; limiting age-appropriate instrumental ADL **Instrumental ADL** includes: preparing meals, shopping for groceries or clothes, using the telephone, managing money, etc.

**Grade 3 -** Severe or medically significant but not immediately life-threatening; hospitalization or prolongation of hospitalization indicated; disabling; limiting self-care ADL. **Self-care ADL** includes: bathing, dressing and undressing, feeding self, using the toilet, taking medications, and not bedridden.

**Grade 4** - Life-threatening consequences; urgent intervention indicated.

**Grade 5** - Death related to AE

| **Opioid Adverse Events** | **Grade** | | | | |
| --- | --- | --- | --- | --- | --- |
|  | 1 | 2 | 3 | 4 | 5 |
| Pruritus/ itching | Mild or localized ; topical intervention indicated | Widespread and intermittent; skin changes from scratching (e.g. Oedema, papulation, excoriations, lichenification, oozing/crusts); oral intervention indicated; limiting instrumental ADL | Widespread and constant; limiting self-care ADL or sleep; systemic corticosteroid or immunosuppressive therapy indicated | - | - |
| Dry mouth | Symptomatic (dry or thick  saliva) without significant  dietary alteration;  unstimulated saliva flow  >0.2 ml/min | Moderate symptoms; oral intake alteration  (e.g., copious  water, other lubricants,  diet limited to purees  and/or soft, moist foods);  unstimulated saliva  0.1 to 0.2 ml/min | inability to adequately  aliment orally;  tube feedings or TPN  indicated; unstimulated  saliva <0.1 ml/min | - | - |
| Nausea | Loss of appetite without  alteration in eating habits | Oral intake decreased  without significant weight  loss, dehydration or  malnutrition | Inadequate oral caloric or  fluid intake; tube  feedings, TPN, or hospitalisation indicated | - | - |
| Confusion | Mild disorientation | Moderate disorientation,  Limiting instrumental ADL | Severe disorientation; limiting self-care ADL | Life-threatening consequences; urgent intervention indicated | - |
| Vomiting | Intervention not indicated | Outpatient IV hydration; medical intervention indicated | Tube feeding, TPN, or hospitalization indicated | Life-threatening  consequences | Death |
| Constipation | Occasional or intermittent  symptoms; occasional  use of stool softeners,  laxatives, dietary  modification, or enema | Persistent symptoms with  regular use of laxatives  or enemas; limiting instrumental ADL | Limiting self-care ADL; obstipation  with manual evacuation  indicated | Life-threatening  consequences (e.g.,  obstruction, toxic  mega colon); urgent intervention indicated | Death |
| somnolence | Mild but more than usual drowsiness or sleepiness | Moderate sedation limiting instrumental ADL | Obtundation or stupor | Life-threatening consequences; urgent intervention indicated | Death |
| Hallucinations | Mild hallucinations (e.g. Perceptual distortions) | Moderate hallucinations | Severe hallucinations; hospitalization not indicated | life-threatening consequences, threats of harm to self or others; hospitalization indicated | Death |
| Apnoea/ Respiratory Depression | - | - | Present; medical intervention indicated | Life-threatening respiratory or hemodynamic compromise; intubation or urgent intervention indicated | Death |
| Others, please specify and grade |  |  |  |  |  |
